# Communicating telecom fraud risk in anti-telefraud messages: The effects of metaphorical frames on attitudes

**DOI:** 10.3389/fpsyg.2022.1093933

**Published:** 2023-01-16

**Authors:** Mengna Liu, Jinshi Chen

**Affiliations:** Center for Linguistics and Applied Linguistics, Guangdong University of Foreign Studies, Guangzhou, China

**Keywords:** telecom fraud, metaphorical framing, war metaphor, disease metaphor, risk communication, anti-telefraud messages

## Abstract

**Introduction:**

With the frequent occurrences of telecom fraud crimes in China, it is very necessary and urgent to carry out effective anti-telefraud risk communication. In the present study, we investigated the role of metaphorical framing in shaping people’s attitudes toward telecom fraud in anti-telefraud messages through two experiments.

**Methods:**

Participants (*N* = 547, Experiment 1; *N* = 604, Experiment 2) were randomly assigned to war-framing, disease-framing, or issue-framing conditions. They were asked to read anti-telefraud messages where metaphorical frames were realized through multiple metaphorical expressions in Experiment 1 and relatively shorter messages where metaphorical frames were only instantiated through one metaphorical word in Experiment 2.

**Results:**

The results showed that participants without prior experience with telecom fraud perceived severity as significantly higher in the war-framing condition than in the issue-framing condition. Besides, the framing effects were only detected in Experiment 2 where the short anti-telefraud message with limited metaphorical information was provided.

**Discussion:**

The study reveals that even one metaphorical word is sufficient to build a framework for thinking about complex concepts, like telecom fraud, and prior experience with a certain risk can serve as a moderator of metaphorical framing on people’s risk perceptions. It is also found that the effectiveness of metaphors may be more salient in the genres of a short length such as anti-telefraud banners. The study can shed light on public legal educators whose job is to use effective ways to communicate telecom fraud risk to citizens.

## 1. Introduction

Metaphors pervade natural discourse to discuss a wide variety of subjects. They are not merely the “rhetorical flourish” that enlivens the discourse; instead, according to Conceptual Metaphor Theory, they act as a cognitive mechanism that prompts people to draw on the knowledge of the concrete concept to interpret the target issue (Lakoff and Johnson, 1980, p. 3). As a fundamental cognitive means in our conceptual system, metaphors help us make sense of elusive things, such as electricity (Gentner and Gentner, 1983), economics (Henderson, 1982), medicine (Coulehan, 2003), and politics (Lakoff, 2008).

Importantly, an extensive body of work suggests that metaphor can guide thought and influence our reasoning about social reality, such as cancer (Hendricks et al., 2018), immigration (Brown et al., 2019), crime (Thibodeau and Boroditsky, 2011) and natural disaster (Hauser and Fleming, 2021). When one comprehends an issue that is metaphorically framed, a conceptual metaphor is activated. Metaphors work in part by activating a conceptual schema connected with the source domain, which prompts people to construct a representation of and subsequently draw inferences about the target domain (for reviews, see Thibodeau et al., 2019). More specifically, metaphor use involves transferring knowledge of a familiar and concrete source in a way that underscores some target features and de-emphasizes others, guiding people to bring their target attitudes consistent with the source knowledge (Lakoff and Johnson, 1980; Thibodeau et al., 2019). For example, metaphorically framing a hurricane as an antagonist increases participants’ forecast of the number of homes destroyed, lives lost, and days without power caused by the hurricane, compared to the literal framing condition. In this case, because antagonists are dangerous, antagonist framing of a hurricane confers such qualities to the hurricane, increasing forecasts of its severity (Hauser and Fleming, 2021).

In this article, we examine the role of metaphor in risk communication about telecom fraud. Telecom fraud refers to a crime in that criminals make up false information, set up scams, and carry out remote and non-contact fraud on victims through telephone, network and SMS, and other telecom tools to induce the victims to make payments or transfer money to the criminals (O’Brien, 1998). In China, telecom fraud crimes have increased in recent years due to factors such as the development of telecommunication technology, and people’s widespread and daily use of mobile devices (Wu, 2015). The recent frequent occurrences of the crimes represent a severe case that has led to economic concerns on a nationwide scale because of its potentially severe effects, including huge loss of money. People, who are easily targeted by doorstep criminals and vulnerable to exploitation, are those lacking anti-telefraud awareness, i.e., the awareness of acting against telecom fraud (Li and Li, 2019). Underestimating telecom fraud risk, they often assume that telecom fraud is far away from their lives and they will never be defrauded. As such, the promotion of people’s anti-telefraud awareness is key to the success of anti-telefraud activities (Yuniarti and Ariandi, 2017).

Developing high-quality and efficient risk communication about telecom fraud is essential for promoting anti-telefraud awareness and preventing economic loss. Understanding the features of persuasive messaging, which promote risk awareness and encourage the adoption of protective behaviors against telecom fraud, is especially important when the telecom fraud interception system is not highly effective, as is the current case for China. Since various online and offline media play a significant role in anti-telefraud publicity, it is useful to study how messaging, including messages framed with colorful metaphors, influences our reasoning about telecom fraud in risk communication. As conceptual metaphors may guide people’s thinking, they provide anti-telefraud practitioners with a possible approach to raising people’s anti-telefraud awareness and improving the efficiency and persuasiveness of anti-telefraud messages.

To gain a better insight into whether and how metaphorical information works in anti-telefraud messages, we ran two experiments to investigate the effectiveness of metaphors in shaping people’s attitudes toward telecom fraud, including risk perceptions, behavioral intentions, and policy support. Specifically, we focused on the metaphors of disease and war, which recent research suggests may enhance risk perceptions toward societal issues (Flusberg et al., 2017; Keefer et al., 2020). In addition, we explored whether the potential metaphorical framing effects in the context of anti-telecom communication would depend on metaphor and information amount in anti-telefraud messages, a key factor we need to consider when public legal educators design anti-telefraud messages.

Furthermore, we considered the potential role of people’s prior experience with telecom fraud in the two experiments. Research suggests that the persuasive impact of metaphorically framed messages may only appeal to a specific group of people and the effects depend on people’s prior knowledge (Landau et al., 2014). That is, metaphor can affect reasoning only when people feel uncertain about the target issues (Landau et al., 2014). Thus, one potential boundary condition on metaphorical framing effects is people’s prior experience with telecom fraud in anti-telefraud risk communication, as their vivid experience may provide them with pre-existing background knowledge and deep-seated beliefs, and moderate the persuasive effects of metaphorical anti-telefraud messages.

## 2. Metaphorical framing

Our starting point is the Conceptual Metaphor Theory’s claim that metaphors are not just linguistic packages of information; they might transfer conceptual content as well (Lakoff and Johnson, 1980, p. 3). This theory posits that metaphors serve as a cognitive tool that people can draw on to understand a concept (target domain) in terms of a superficially unrelated concept (source domain; for overviews, see Lakoff and Johnson, 1980; Gibbs, 1994; Kövecses, 2010). The target domain involves typically novel, abstract or complex concepts such as social-political issues (e.g., immigration) or intangible things (e.g., love, depression). The source domain is relatively more concrete, familiar and easier to understand, referring to embodied experience (e.g., experience of movement, space, feeling cold or hot) or familiar scripts (e.g., what do people experience in a journey or war). Metaphors facilitate comprehension of the target by conceptually mapping its features onto analogous features of the source: in this way, metaphors transfer familiar and concrete source knowledge to support interpretations of the target, which highlights the entailments of the target in line with the source. For example, people commonly understand the elusive process of life metaphorically in terms of a physical journey (e.g., my life is on the right track). The mapping created by the metaphor life is a journey transfers a conceptual template that life choices are branching paths, difficulties are obstacles and life progress is forward movement, emphasizing the similarities between life and journey.

Serving as frames or existing cognitive schemas that help interpret information, the more concrete source concepts can highlight similarities between the two domains while downplaying dis-similarities (Lakoff and Johnson, 1980, p. 10), which makes metaphor the framing device and reasoning device *par excellence*. As argued by Burgers et al. (2016), metaphors can achieve one or more of the functions of framing as proposed by Entman (1993, p. 52), “to frame is *to select some aspects of a perceived reality and make them more salient in a communicating text, in such a way as to promote a particular problem definition, causal interpretation, moral evaluation, and/or treatment recommendation for the described item*” (italics in original). For example, thinking about a wildfire has been found to elevate people’s perceived loss and increase people’s willingness to evacuate when the wildfire is metaphorically framed as a monster, compared to when the wildfire is literally depicted (Matlock et al., 2017). In this situation, a monster, which refers to wildfire, is a danger (problem definition) that causes serious problems (causal interpretation), is difficult to control (problem evaluation), and requires action to stop it from harming society and citizens (treatment recommendation).

In line with Conceptual Metaphor Theory, a growing body of research has shown evidence that metaphors can affect how people reason on the issue that is addressed, and hence steer recipients’ opinions more in line with the metaphorical frame (for reviews, see Thibodeau et al., 2017, 2019; Van Stee, 2018). Because metaphors facilitate comprehension, it should not come as much of a surprise that they are frequently used to symbolize intangible aspects of societal issues and potential responses to those issues (Keefer and Landau, 2016). Thibodeau and Boroditsky (2011) explored how metaphors crime is a disease and crime is a wild beast influenced the way that people reasoned about complex issues, such as generating different solutions to a city’s crime problem and foraging for further information about them. Participants in the crime-as-a-beast condition chose enforcement-oriented solutions more frequently than those in the crime-as-a-virus condition. Contrastingly, participants in the virus condition were more inclined to favor reform-oriented solutions, such as prevention and education reform. They found that even the limited metaphorical information (*via* a single word) could powerfully influence how people attempted to solve social problems like crime and how they gathered information to make “well-informed” decisions. Christmann and Göhring (2016) replicated Thibodeau and Boroditsky’s study (2011) in the German language, concluding that metaphors do indeed frame reasoning. Thibodeau and Boroditsky (2013) further found that the crime is a disease and crime is a wild beast metaphors could influence people’s reasoning even when they had a set of options available to compare and select among, which suggests metaphors can influence not just what solution comes to mind first, but also which solution people think is best. Interestingly, few participants thought the metaphor was crucial in their decision, which displays that the effects of metaphor framing are predominantly covert.

When it comes to conveying social, health, or environmental risks, metaphors are useful communicative tools in that they enable speakers to describe risk issues in terms of simple and more familiar domains (e.g., Vasquez et al., 2014; Flusberg et al., 2017; Matlock et al., 2017). Specifically, metaphors help people establish common ground between the danger conveyed by the source image and the target risk, and efficiently transfer a range of structured attitudes. Supporting evidence suggests that framing a risk with a metaphor can also influence people to reason about the risk in a metaphor-congruent fashion. To take a few recent examples: (1) when the flood and hurricane were metaphorically framed as an antagonist, people were likely to forecast more damage caused by the natural disaster, compared with literal or vehicle framing conditions (Hauser and Fleming, 2021), (2) animalistic descriptions of criminal acts resulted in significantly higher perceived risk of recidivism related to perpetrators and accordingly, harsher punishment for them (Vasquez et al., 2014), and (3) when the influenza was metaphorically compared to a wild animal attacking one’s health, a weed growing inside one’s body or an invading army, people were more likely to have intentions to get a flu shot (Scherer et al., 2015). In related studies, metaphors influence attitudes toward risk issues, including cancer (Hendricks et al., 2018), COVID-19 (Panzeri et al., 2021), immigration (Brown et al., 2019), climate change (Flusberg et al., 2017), and wildfire (Matlock et al., 2017).

Especially, the war metaphor (e.g., “war on cancer,” “climate change is war”) and the disease metaphor (e.g., “plagiarism is a disease,” “euro crisis is a disease,” “immigration is a disease”) are widely adopted in risk communication (Flusberg et al., 2017; Hendricks et al., 2018; Brown et al., 2019; Joris et al., 2019; Keefer et al., 2020), because war and disease are easily-understood concepts with well-defined attributes. The effectiveness mainly depends on the negative scenarios with strong risk conveyed by the two metaphorical frames, by seeing an abstract problem in terms of a concrete threat, i.e., war or disease. For example, for the war frame, Flusberg et al. (2017) found that the war metaphor shaped people’s attitudes toward climate change, which improved the perceived urgency and willingness to curb climate change, relative to the race metaphor and the literal version. For the disease frame, the findings of Keefer et al.’s (2020) study showed that framing plagiarism with the disease metaphor led students to perceive plagiarism as a more severe problem and as a result, they were more supportive of anti-plagiarism policies. The results of the experiments provide evidence that war and disease metaphors have far-reaching implications for the perception and evaluation of societal issues in risk communication. It is suggested that war and disease metaphors can activate conceptual schemas that are used to reason about a target domain and elicit cognitive or affective responses that influence reasoning about risks. The two metaphors for societal issues cause people to see the issues as riskier and more pressing.

However, even though metaphors show strong effects in many risk communication and other field studies, some research suggests that metaphorical framing effect can be conditional and individual differences can modify the direction and/or strength of the message’s framing effects (e.g., Landau et al., 2014; Thibodeau and Boroditsky, 2015; Brown et al., 2019). Some work shows that people’s prior knowledge serves as one key individual difference that matters to the persuasive effects of metaphorical framing (e.g., Landau et al., 2014; Thibodeau and Boroditsky, 2015). More specifically, the research posits that when people feel certain about their knowledge of the target, metaphors become less influential than when people feel uncertain about it. For instance, Landau et al. (2014) found that metaphorically framing corporate bankruptcy as a car crash prompted participants to attribute more blame to the CEO of the company, relative to the non-metaphorical condition. However, the metaphorical framing effects were diluted for participants who felt confident in their prior knowledge of corporate bankruptcies, compared with those who felt less confident. Similarly, another study also showed that metaphors were less persuasive for people who held deep-seated attitudes about how to best deal with a crime problem than those who do not (Thibodeau and Boroditsky, 2015). These studies suggest that people’s knowledge of the target domain must be somewhat malleable for a metaphor to influence them and deep-seated beliefs about a target domain can “interfere with a metaphorical mapping” (Thibodeau et al., 2017, p. 4).

## 3. The current study

Drawing from the insights from metaphorical framing research, the present study investigated whether conceptual metaphors influenced people’s reasoning about telecom fraud in anti-telefraud risk communication. Specifically speaking, we focused on the war metaphor and disease metaphor, which are two prevalent frames for communicating risk issues and are commonly used to confer danger to the target topics in risk communication.

War and disease metaphors are pervasive in anti-telefraud discourse to metaphorically describe telecom fraud, as in “阻断电信诈骗入侵校园 (Blocking telecom fraud from invading the campus)” (Sohu, 2021) and “网络诈骗犯罪仍是‘社会毒瘤’ (Internet fraud is still a ‘social cancer’)” (Xinhua Net, 2019). The war metaphor characterizes telecom fraud as enemies that lurk around citizens and invade their lives. As such, measures need to be taken to build the defense line and combat the enemies, as in “罗甸剬安多举措打防电信诈骗战果明显 (Luodian public security bureau has taken many measures to defend and fight against telecom fraud, with positive results of war)” (Tencent, 2021). The disease metaphor characterizes telecom fraud as diseases or disease-related concepts, such as “stubborn disease,” “malignant tumor,” and “virus.” As such, a remedy for telecom fraud is needed such as the inoculation of the vaccine or preventive injection, as in “廉江剬安为群众接种 ‘反诈疫苗’, 打好防骗预防针 (Lianjiang public security bureau inoculates the masses with ‘anti-fraud vaccine’, a good preventive shot against fraud)” (Netease, 2021). Because war and disease metaphors are commonplace in discussions about telecom fraud, it is important to know whether such metaphors can serve as a cognitive tool that can influence people’s reasoning about telecom fraud.

To remind people to avoid telecom fraud, the use of war or disease metaphors, which evoke domains that elicit perceived risk, may be a productive strategy. Human beings have evolved to stay away from things they consider to be pathogenic and dangerous to reduce the chance of infection and injury (Kagan, 1996; Murray and Schaller, 2016). Thus, language describing telecom fraud systematically as a disease or an enemy could cause perceivers to transfer the perceived risk to a new domain. We referred to Lu and Schuldt’s (2018) research and decomposed the multi-faceted concept of risk perceptions as perceived severity (judgments of seriousness), perceived susceptibility (likelihood of being affected), [negative affect (affect response to risk) Cummings et al., 1978; Janssen et al., 2011)]. The war and disease metaphors both have a strongly negative valence, which conveys danger and severity and potentially elicits fear, disgust, anger, and anxiety in human emotions. Thus, we predicted that, compared with those who read the materials with the non-metaphorical frame, participants in war-framing and disease-framing conditions, would have stronger risk perceptions, specifically speaking, perceived severity, perceived susceptibility, and negative affect toward telecom fraud, relative to the literal version.

Some work suggests that individuals are more willing to act and propose responses that align with the salient concepts offered by metaphors when metaphors prime individuals to perceive a diffuse abstract problem as a concrete threat (e.g., Thibodeau and Boroditsky, 2015; Keefer et al., 2020). Thus, war-fighting and disease-preventing narratives potentially tap into people’s expectations of solving problems and willingness to engage in anti-telefraud activities, as Yanow (2008, p. 237), argues that metaphor is both a “model of” a phenomenon and a “model for” actions concerning that phenomenon. Thus, we predicted that participants exposed to metaphorical frames would show a stronger motivation in their behavioral intentions. Besides, we also predicted that participants exposed to metaphorical frames would support more in terms of the anti-telefraud policy proposed by the government and consider it more of a priority.

As illustrated in the previous sections, the metaphorical framing effects can vary with individual-level factors and people’s prior knowledge of the target domain must be somewhat malleable for metaphors to influence them (Thibodeau et al., 2017). Therefore, we considered the potential role of people’s prior experience with telecom fraud in metaphorical framing effects, as their vivid experience may lead them to form pre-existing knowledge and entrenched mental models about the telefraud topic. As deep-seated beliefs about a topic can make a person less amenable to persuasion by metaphor (Landau et al., 2014), we might find that the metaphorical framing effects are smaller for people who have prior experience with telecom fraud, and are more likely to form unambiguous judgments about the telefraud topic, than for people who do not have such vivid experience. Thus, we predicted that metaphorical framing effects would be greater for participants without prior experience with telecom fraud than for those with such prior experience.

Of note, we operationalized prior experience as having been defrauded, or having at least one family member or close friend who has been defrauded, including both direct and indirect experience. We added indirect experience to the variable of participants’ prior experience, given that their vicarious experience from close relationships could also influence people’s pre-existing attitudes toward telecom fraud through victim-telling or direct observation.

Besides, in the current study, we also investigated the role of metaphor and information amount in metaphorical framing effects, a key factor we need to consider in designing anti-telefraud messages. Two experiments were conducted to examine the framing effects of metaphor in anti-telefraud discourse when metaphorical frames were realized through various metaphorical expressions and linguistic relations in a relatively longer text (i.e., the greater number of words and the greater amount of information), or just through limited metaphorical information, such as one metaphorical word in a short text, respectively.

We used a common type of telecom fraud, i.e., loan fraud, as an example to organize the experimental materials. Loan fraud was chosen as an example, as loan fraud has become an increasingly prominent issue in recent years. More people in China are considering enjoying petty services, for instance, consumer credit from apps like Alipay, with the acceptance of the popular concept of “buy-now-pay-later” (Huang, 2021, p. 2). Some people also seek relatively large loan services for making up for the shortage of funds, such as in investment or buying houses. The ensuing loan fraud that frequently appears nowadays deserves a high priority in anti-telefraud campaigns.

## 4. Experiment 1: Can war metaphor and disease metaphor influence people’s attitudes toward telecom fraud?

### 4.1. Method

#### 4.1.1. Design

Experiment 1 aimed to test whether there were differences in people’s attitudes about telecom fraud (including risk perceptions, behavioral intentions, and policy support) between the subjects exposed to the war frame, disease frame, and issue frame. The metaphorical frames in the experiments were realized by a variety of metaphorical expressions and linguistic relations.

#### 4.1.2. Participants

Participants were recruited through a post on Wenjuanxing, the Chinese version of Mechanical Turk, and were paid 2 yuan for completing the experiment. Completed questionnaires were obtained from exactly 547 participants. Submissions were excluded from the study as follows: (a) 33 respondents refused to participate in the task, (b) 11 respondents spent <100 s finishing the whole task, (c) 19 respondents failed to correctly answer the attention-checking question, suggesting low attention to the stimulus materials, (d) 2 questionnaires were submitted repeatedly from the same IP address, and (e) 12 respondents who aged below 18 were excluded. The final samples consisted of 470 participants. These individuals spent a mean of 354.90 s (SD = 378.34) finishing the task. The sample was 67.02% female and 32.98% male with a mean age of 37.63 years (SD = 9.11). The median highest level of education attained was a bachelor’s degree. People with prior experience with telecom fraud account for 32.98%.

#### 4.1.3. Stimulus materials and procedure

Participants were randomly assigned to one of three experimental conditions, i.e., war frame, disease frame, or issue frame. The experiment was conducted online using Wenjuanxing. After giving informed consent, participants read a fabricated message released on Weibo, a Chinese social media platform similar to Twitter. They then completed a questionnaire on the next page. Time spent on finishing the questionnaire was covertly recorded. The back button was forbidden to prevent participants from returning to the Weibo message while completing the questionnaire.

Participants first read a brief Weibo message about the current situation of loan fraud in China that varied with the experimental conditions. Each message was titled “Beware of the enemy of loan fraud,” “Beware of the virus of loan fraud” and “Beware of the issue of loan fraud.” Participants in this experiment were randomly allocated to one of these three frames. The frame was presented as the title of the articles and then was extended throughout the description. For example, in the war-framing condition, participants read statements about how the enemy of loan fraud were seeking to attack people and that we needed to use weapons to combat him. In the disease-framing condition, participants read about how the virus of loan fraud was seeking to affect people and that we needed to use vaccines to prevent it. In the issue-framing condition, participants read about how the issue of telecom fraud troubled us and that we needed to solve this social problem. Aside from the metaphorical contents, we kept the linguistic forms used in the Weibo message identical, including the same sentence structures, for example, “Loan fraud is the enemy we must combat.”, “Loan fraud is the virus we must clean.”, and “Loan fraud is the issue we must solve.” The illustrative language manipulations in the three passages were presented in Table 1. No words were emphasized in the original materials. The number of words in three conditions is 205 words, 210 words, and 212 words, respectively.

**Table 1:** Illustrative language manipulations with translations in Experiment 1 (Differences across conditions are underlined).

Issue frame	War frame	Disease frame
当心贷款诈骗问题 (Beware of the issue of loan fraud)	当心贷款诈骗敌人 (Beware of the enemy of loan fraud)	当心贷款诈骗病毒 (Beware of the virus of loan fraud)
近年来，随着电信网络的发展，贷款诈骗这个问题在我们身边出现。 In recent years, with the development of telecommunication networks, the issue of loan fraud has appeared around us.	近年来，随着电信网络的发展，贷款诈骗这群敌人在我们身边发起了侵袭。 In recent years, with the development of telecommunication networks, the enemy of loan fraud has launched an attack on our side.	近年来，随着电信网络的发展，贷款诈骗这种病毒在我们身边传播开来。 In recent years, with the development of telecommunication network, the virus of loan fraud has spread around us.
贷款诈骗可能存在于很多地方，如手机短信平台、社交媒体、虚拟网站等等。 Loan fraud may exist in many places, such as SMS platforms, social media, virtual websites, and so on	贷款诈骗可能埋伏在很多地方，如手机短信平台、社交媒体、虚拟网站等等。 Loan fraud may be lurking in many places, such as SMS platforms, social media, virtual websites, and so on	贷款诈骗可能扩散到了很多地方，如手机短信平台、社交媒体、虚拟网站等等。 Loan fraud may spread to many places, such as SMS platforms, social media, virtual websites, and so on.
若不小心，则可能会遭到贷款诈骗的困扰。 If we are not careful, we may be troubled by loan fraud.	若不小心，则可能会遭到这群敌人的攻击。 If we are not careful, we may be attacked by the enemy.	若不小心，则可能会遭到这种病毒的伤害。 If we are not careful, we may be affected by the virus.
为了减少诈骗，我们需要找到能有效解决贷款诈骗问题的办法。 To reduce fraud, we need to find an effective solution to loan fraud.	为了重挫敌人，我们需要拿出能有效打击贷款诈骗敌人的武器。 To defeat the enemy, we need to come up with weapons that can effectively combat loan fraud.	为了遏制病毒，我们需要开发能有效免疫贷款诈骗病毒的疫苗。 To contain the virus, we need to develop a vaccines that can effectively immunize against loan fraud.
反诈在行动，电信网络贷款是我们要解决的问题。 Anti-telefraud activities are in action, and loan fraud is the problem we must solve.	反诈在行动，电信网络贷款是我们要击败的敌人。 Anti-telefraud activities are in action. Loan fraud is the enemy we must defeat.	反诈在行动，电信网络贷款是我们要清除的病毒。 Anti-telefraud activities are in action. Loan fraud is the virus we must eliminate.

After reading the Weibo message, participants were asked to make judgments about risk perceptions. Specifically, for perceived severity, participants indicated how severe they believed loan fraud crimes have been on a scale ranging from 1 = Not severe at all to 11 = Extremely severe. For perceived susceptibility, participants indicated their perceived likelihood of becoming encountered with loan fraud personally on a scale ranging from 1 = Not likely at all to 11 = Extremely likely. For negative affect, participants indicated among a battery of negative emotion items how much they felt each of the listed emotions when thinking about loan fraud (1 = None of this feeling to 11 = A lot of this feeling): fear, disgust, anger, and anxiety. These four emotion items were averaged into a composite scale representing negative affect (Cronbach’s *α* = 0.85).

Then we investigated participants’ attitudes toward anti-telefraud policy support. Participants reported how much they thought the government should prioritize curbing loan fraud crime on a scale ranging from 1 = Should not be a priority at all to 11 = Should be the top priority.

Participants were then asked to read a list of fraud prevention behaviors and indicated their willingness of adopting these behaviors in the following year (1 = Not likely at all to 11 = Extremely likely).

1. Would you be willing to download the app designed for anti-loan fraud activities?
2. Would you be willing to receive SMS or email about anti-loan fraud messages regularly?
3. Would you be willing to read or watch public articles or videos about anti-loan fraud activities?
4. Would you be willing to attend the public lecture on anti-loan fraud activities? These items were averaged to create a composite scale of behavioral intentions (Cronbach’s *α* = 0.83).

People were also asked a question about their prior experience with telecom fraud, i.e., whether they themselves, their family members or close friends had been defrauded before or not. The prior experience with telecom fraud was included as a categorical predictor in the following behavioral analysis to test whether the effects of the experimental manipulations were moderated by participants’ prior experience with telecom fraud.

Then, one attention check question was included to assess whether participants paid attention to the experimental materials. The question asked participants to summarize at least one keyword about the main idea of the passage. Finally, demographic questions about gender, age, and educational background were asked. The data for the experiments are available in Supplementary material.

### 4.2. Results

To test whether metaphors influenced risk perceptions, behavioral intentions, and policy support, a series of two-way ANOVAs were conducted that compared the extent to which participants in each condition were affected by each framing.

#### 4.2.1. Perceived severity

There was no statistical significance of the main effect of experimental conditions, *F*(2,464) = 1.127, *p* = 0.325, but the main effect of prior experience on perceived severity displayed a trend, *F*(1,464) = 3.164, *p* = 0.076, *η_p_*^2^ = 0.007, such that participants with prior experience (*M* = 9.10, SD = 1.92) perceived severity higher than those without prior experience (*M* = 8.72, SD = 2.06). There was no significant interaction effect between experimental conditions and prior experience, *F*(2,464) = 1.087, *p* = 0.338, on perceived severity.

#### 4.2.2. Perceived susceptibility

Turning to perceived susceptibility, a trend was displayed on the main effect of the experimental conditions, *F*(2,464) = 2.765, *p* = 0.064, *η_p_*^2^ = 0.012. A *post-hoc* test with Bonferroni-corrections showed there was a trend that the war-framing message (*M* = 7.67, SD = 3.18) resulted in more perceived susceptibility than the literal message (*M* = 6.95, SD = 3.02), *p* = 0.098. There were no main effect of prior experience, *F*(1,464) = 2.376, *p* = 0.124, or significant interaction effects, *F*(2,464) = 1.033, *p* = 0.357.

#### 4.2.3. Negative affect

Concerning negative affect, the main effect of prior experience with telecom fraud was observed, *F*(1,464) = 7.040, *p* = 0.008 < 0.05, *η_p_*^2^ = 0.015. Descriptive statistics showed that the participants with prior experience with telecom fraud (*M* = 8.24, SE = 2.37) had more negative affect than those without prior experience (*M* = 7.53, SE = 2.63). We did not find a main effect of the experimental conditions, *F*(2,464) = 0.601, *p* = 0.548. There was also no interaction effect on participants’ negative affect, *F*(2,464) = 0.053, *p* = 0.948.

#### 4.2.4. Behavioral intentions

There were no main effects of framing conditions, *F*(2,464) = 1.181, *p* = 0.308, and prior experience, *F*(1,464) = 0.978, *p* = 0.323, on participants’ behavioral intentions. Besides, we did not find any interaction effect on participants’ behavioral intentions, *F*(2,464) = 0.912, *p* = 0.403.

#### 4.2.5. Policy support

There were no main effects of framing conditions, *F*(2,464) = 1.969, *p* = 0.141, and prior experience, *F*(1,464) = 0.040, *p* = 0.842, on participants’ policy support. Besides, there was also no interaction effect on participants’ policy support, *F*(2,464) = 0.465, *p* = 0.629.

## 5. Experiment 2: Can limited metaphorical information influence people’s attitudes toward telecom fraud?

### 5.1. Method

#### 5.1.1. Design

Experiment 2 aimed to explore how people’s attitudes about telecom fraud were affected by limited metaphorical information in a short text. In this experiment, only the first sentence of the experimental stimuli contained one metaphorical term, such as the virus/the enemy of loan fraud has appeared around us. The total words of the stimuli for each framing condition were only about one-third of those in Experiment 1.

#### 5.1.2. Participants

Participants in Experiment 2 were recruited through a post on Wenjuanxing and were paid 2 yuan for completing the experiment. Completed questionnaires were obtained from exactly 604 participants. Submissions were excluded from the study: (a) 44 respondents refused to participate in the task, (b) 40 respondents spent <80 s finishing the whole task, (c) 36 respondents failed to correctly answer the attention-checking question, suggesting low attention to the stimulus materials, and (d) 4 respondents who aged below 18 or did not fill in his/her exact age were excluded. The final samples consisted of 482 participants. These individuals spent a mean of 265.75 s (SD = 238.33) finishing the task. The sample was 63.07% female and 36.93% male with a mean age of 31.79 years (SD = 11.69). The median highest level of education attained was a bachelor’s degree. People with prior experience with telecom fraud account for 37.76%.

#### 5.1.3. Stimulus materials and procedure

In Experiment 2, we changed the representation of metaphorical frames and the message length in the stimulus materials to further examine the role of metaphor in reasoning. In this experiment, we just used only one word to instantiate the metaphorical frames. The illustrative language manipulations in the three passages were presented in Table 2. Besides, the information about loan fraud in the stimuli was reduced to two sentences. The number of words in three conditions is all 70 words across three conditions. The experimental procedures were the same as those in Experiment 1.

**Table 2:** Illustrative language manipulations with translations in Experiment 2 (Differences across conditions are underlined).

Issue frame	War frame	Disease frame
近年来，随着电信网络的发展，贷款诈骗这个问题已经出现在了我们的身边。 In recent years, with the development of telecommunication networks, the issue of loan fraud has appeared around us.	近年来，随着电信网络的发展，贷款诈骗这群敌人已经出现在了我们的身边。 In recent years, with the development of telecommunication networks, the enemy of loan fraud has appeared around us.	近年来，随着电信网络的发展，贷款诈骗这种病毒已经出现在了我们的身边。 In recent years, with the development of telecommunication networks, the virus of loan fraud has appeared around us.

### 5.2. Results

#### 5.2.1. Perceived severity

No main effects were found for framing conditions, *F*(2,476) = 1.798, *p* = 0.167, and previous experience, *F*(1,476) = 1.449, *p* = 0.229, on perceived severity. However, the interaction effect between framing conditions and prior experience displayed a trend, *F*(2,476) = 2.661, *p* = 0.071, *η_p_*^2^ = 0.011. Post-host tests with Bonferroni-corrections showed that for people without prior experience, the war-framing message (*M* = 9.10, SD = 1.85) aroused higher perceived severity than the issue-framing message (*M* = 8.41, SD = 1.75), *p* = 0.017. Besides, in the issue-framing message, people with prior experience (*M* = 9.13, SD = 1.45) perceived loan fraud as significantly more severe than people without prior experience (*M* = 8.41, SD = 1.75), *p* = 0.009.

#### 5.2.2. Perceived susceptibility

There was no main effect of framing conditions, *F*(2,476) = 1.225, *p* = 0.295, and no significant interaction effect, *F*(2,476) = 0.976, *p* = 0.378, on perceived susceptibility, but we found a significant effect of prior experience with perceived susceptibility, *F*(1,476) = 6.631, *p* = 0.01, *η_p_*^2^ = 0.014. People with prior experience with telecom fraud (*M* = 8.01, SD = 2.42) perceived loan fraud as significantly more susceptible than those without prior experience with telecom fraud (*M* = 7.37, SD = 2.75).

#### 5.2.3. Negative affect

Turning to negative affect, no main effect of experimental conditions was found on negative affect, *F*(2,476) = 0.325, *p* = 0.723. However, results revealed a significant main effect of prior experience, *F*(1,476) = 4.302, *p* = 0.039, *η_p_*^2^ = 0.009, such that people with prior experience with telecom fraud (*M* = 7.78, SD = 2.17) perceived more negative affect than people without prior experience with telecom fraud (*M* = 7.30, SD = 2.44). The main effect, however, was qualified by the trend of a two-way interaction, *F*(2,476) = 2.387, *p* = 0.093, *η_p_*^2^ = 0.01. A post-host test with Bonferroni-corrections showed that only in the issue-framing condition, people with prior experience with telecom fraud rated the measure of negative affect significantly higher than people without prior experience with telecom fraud (*p* = 0.003).

#### 5.2.4. Behavioral intentions

With respect to behavioral intentions, no main effects of framing conditions, *F*(2,476) = 1.014, *p* = 0.364, and prior experience, *F*(1,476) = 0.119, *p* = 0.731, were found on people’s behavioral intentions. There was also no interaction effect between framing conditions and prior experience with people’s behavioral intentions, *F*(2,476) = 0.020, *p* = 0.980.

#### 5.2.5. Policy support

Regarding policy support, no significant effect of framing conditions, *F*(2,476) = 0.410, *p* = 0.664, and no interaction effect between framing conditions and prior experience, *F*(2,476) = 0.821, *p* = 0.441, were found on people’s policy support. However, there was a significant effect of prior experience with people’s policy support, *F*(1,476) = 6.126, *p* = 0.014. People with prior experience with telecom fraud (*M* = 9.54, SD = 1.37) rated the priority of anti-loan fraud activity as significantly higher than people without prior experience with telecom fraud (*M* = 9.18, SD = 1.67).

## 6. Discussion

The two experiments presented in this article explored the cognitive consequences of war and disease metaphors on people’s attitudes toward telecom fraud in two different metaphorical information contexts. Participants read a passage about loan fraud, either framed as a/an “enemy,” “virus” or “issue.” They then responded to questions that probed their attitudinal landscape about loan fraud, including risk perceptions (which are composed of perceived severity, perceived susceptibility, and negative affect), behavioral intentions, and policy support. In Experiment 1, an overall significant effect on attitudes toward loan fraud was not found. In Experiment 2, we found that for participants without prior experience, the war-framing message generated more perceived severity of loan fraud than the issue-framing message.

Although the metaphorical framing effect is chiefly limited to the perceived severity of loan fraud in the war-framing condition of Experiment 2, the result implies, consistent with previous research (Thibodeau and Boroditsky, 2011; Matlock et al., 2017), even one metaphorical word can shape people’s thoughts about a certain risk. It adds to evidence that one metaphorical word is sufficient to build a framework for thinking about complex concepts, like telecom fraud, and support the metaphorical mapping process which makes similarities in relational structure salient, and imbues the target with some of the source’s features (Gentner and Gentner, 1983). This structure mapping process is likely responsible for the higher perceived severity we observed in Experiment 2 when loan fraud was framed as an “enemy,” relative to the literal version.

The present study also contributes to the literature in the metaphor and risk communication research fields in that it testifies that prior experience with the target domain (a certain risk) serves as a moderator of metaphorical framing on people’s risk perceptions. The role of prior experience with telecom fraud was displayed in metaphorical framing effects, as the effects of war framing, which led to higher perceived severity, are limited to the participants without prior experience with telecom fraud. As previous studies argued, participants who have existing knowledge structures on a target are less likely to be influenced by metaphorical frames (Robins and Mayer, 2000), as the frames can only affect participants whose views on a target are somewhat malleable (Landau et al., 2014; Thibodeau and Boroditsky, 2015). Thus, the metaphorical framing effects may be attenuated if the knowledge structure that is related to the severity of telecom fraud is primed, due to participants’ vivid experiences with telecom fraud that helps them to understand the situation (Robins and Mayer, 2000). In contrast, participants without such vivid experiences may be more easily affected and use metaphors to make sense of aspects of risky issues when they are ambiguous about the telefraud topic.

Considering that citizens without first-hand experience with telecom fraud take a large proportion, war-framing messages can be employed to target communities for achieving more effective anti-telefraud publicity by public legal education practitioners. Although no framing effects were detected in the other dimensions of risk perceptions, the observed effects of metaphorical framing on the perceived severity can have significant impacts in anti-telefraud media campaigns where the outcomes are possibly influenced by slim margins (Prentice and Miller, 1993). Especially, the finding of metaphorical framing effects in the short text indicates that the war metaphor can be especially considered to be used in the anti-telefraud banners, a typical short text. Although it is rather naïve to presume that a single media message will directly keep people away from loan fraud, the results revealed that metaphorical wording can be an effective and cost-efficient way of anti-telefraud publicity.

In the study, we detected significant war framing effects in perceived severity only in Experiment 2, but not in Experiment 1. The findings point to the possibility that metaphorical frames may be ineffective at elevating perceived severity when the messages on a risk issue in question already provide a sufficient amount of information on its severity. Thus, the detailed descriptions of loan fraud in Experiment 1 make its severity salient for participants both with prior experience and without it, regardless of the use of metaphors. On the contrary, in the short texts that lack a detailed description to present loan fraud risk, even one metaphorical word has the capacity to highlight its severity, thus displaying the framing effect. This implies that anti-telefraud messages that vary in length in different channels can influence metaphorical framing effects, such that the effects in the banners for anti-telefraud publicity can be more salient than those in other channels, such as leaflets and posters.

No metaphorical framing effects in risk perceptions were found in the disease-framing condition. People’s perception of the metaphors possibly explains why we did not detect disease framing effects but war framing effects in the present study. For example, metaphor novelty can influence the processing fluency of a metaphorical sentence (Pierce and Chiappe, 2008), and perceived aptness has been regarded as a prerequisite for the persuasive effects of metaphorical language (Thibodeau and Durgin, 2011). The use of war metaphor and disease metaphor may vary in the aptness and novelty in anti-telefraud discourse, leading to different framing effect sizes. Nevertheless, we have not been able to establish the extent to which the disease metaphor and the war metaphor used in the anti-telefraud context are perceived as apt and novel. Empirical studies are further needed to test participants’ perceived novelty and aptness of the metaphors.

In the two experiments, we find no significant metaphorical framing effects on policy support and behavioral intentions. Probably, since policy support does not require much personal effort (Lu and Schuldt, 2018), the dimensions of policy support across the experimental conditions all show a very high level (Experiment 1: *M*_overall_ = 9.87, SD_overall_ = 1.75; Experiment 2: *M*_overall_ = 9.32, SD_overall_ = 1.57), with participants’ overall relatively high levels of risk perceptions. Besides, the high level of policy support demonstrates the typical collectivism in Chinese political culture, in which citizens tend to think that government should shoulder a high responsibility for social welfare (Yang et al., 2019). The behavioral intentions also demonstrate a relatively high level across three experimental conditions in two experiments (Experiment 1: *M*_overall_ = 9.09, SD_overall_ = 1.84; Experiment 2: *M*_overall_ = 8.47, SD_overall_ = 1.92). The reason may be that the behaviors listed in the questionnaires do not involve the actual cost of money and are also relatively easy to achieve. In addition, we use the self-reports to measure behavioral intentions, which may not reflect their real behavioral change (Webb and Sheeran, 2006) and thus we do not know whether people will engage in anti-telefraud activities at the high level as reported.

## 7. Implications, limitations, and future directions

The current study carries implications both practically and theoretically. First, the study can shed light on how anti-telefraud practitioners describe telecom fraud in future anti-telefraud activities. The results demonstrate that metaphors are not merely rhetorical devices that can be added to or removed from anti-telefraud messages without affecting people’s perceptions. Designers of anti-telefraud messages can leverage war metaphors to make the severity of telecom fraud salient, and anti-telefraud target group, especially people without prior experience with telecom fraud, can draw on the metaphors to ascertain its severity even through one metaphorical word. Overall, the study suggests that metaphorical information used to discuss telecom fraud deserves consideration in the development of models of effective anti-telefraud risk communication.

Second, the study suggests that anti-telefraud messages that vary in length in different channels can influence the persuasive effects of metaphorical framing, such that the effects in the banners for anti-telefraud publicity can be more salient than those in other channels, such as leaflets and posters. The results further indicate that message characteristics, such as message length and further the amount of risk information, may also affect metaphorical framing effects in risk communication, which needs to be testified in future studies.

Third, the current study contributes to the literature in the metaphor and risk communication research fields in that it testifies that people’s prior experience with a certain risk is an important moderator of the persuasive impact of metaphorical framing on risk perception. To get a better sense of how metaphorical framing works in risk communication, future studies should pay attention to the variable of prior experience with a certain risk in their investigations.

Despite the contributions, the limitations of the present study have to be acknowledged and future directions are discussed below. The present study only focuses on the moderator of prior experience, and only investigates the effects of metaphorical framing on one type of telecom fraud. Future research on additional individual differences and the effects of metaphorical frames on other kinds of telecom fraud should also be investigated.

## 8. Conclusion

The article conducted two experiments to test whether the public’s attitudes toward loan fraud, including risk perceptions, public support, and behavioral intentions, would be influenced by war or disease framings. Five hundred forty-seven and Six hundred and four participants were exposed to either war-framing, disease-framing, or issue-framing messages in the two experiments, respectively. They were asked to read anti-telefraud messages where metaphorical frames were realized through multiple metaphorical expressions and linguistic relations in Experiment 1, and relatively short messages where metaphorical frames were instantiated through one metaphorical word in Experiment 2. We found out that participants without prior experience with telecom fraud perceived severity as significantly higher in the war-framing condition than in the issue-framing condition. Besides, the framing effects were only detected in anti-telefraud messages in a short length. Altogether, the two experiments demonstrated a scenario of the potential effects of metaphorical messages on people’s attitudes in anti-telefraud risk communication. As a result, this study may potentially be useful to public legal educators whose job is to use effective ways to communicate telecom fraud risk to a general audience.

## Data availability statement

The original contributions presented in the study are included in the article/Supplementary material, further inquiries can be directed to the corresponding author.

## Ethics statement

The studies involving human participants were reviewed and approved by Guangdong University of Foreign Studies. The patients/participants provided their written informed consent to participate in this study.

## Author contributions

ML and JC conceived of the initial idea, designed the study, collected the data, and analyzed the data. ML drafted the manuscript. JC revised subsequent versions, proofread the manuscript, and finalized the manuscript for submission as the corresponding author. All authors contributed to the article and approved the submitted version.

## Funding

This paper is a part of the National Social Sciences Research Foundation entitled “A Corpus-based Study of the identification of Telecom Fraud Discourse and its Application (no. 18BYY073).”

## Conflict of interest

The authors declare that the research was conducted in the absence of any commercial or financial relationships that could be construed as a potential conflict of interest.

## Publisher’s note

All claims expressed in this article are solely those of the authors and do not necessarily represent those of their affiliated organizations, or those of the publisher, the editors and the reviewers. Any product that may be evaluated in this article, or claim that may be made by its manufacturer, is not guaranteed or endorsed by the publisher.

## Supplementary material

The Supplementary material for this article can be found online at: https://www.frontiersin.org/articles/10.3389/fpsyg.2022.1093933/full#supplementary-material
